# Growth type and relative condition factor as a function of the body shape of deep-water crustaceans in the Colombian Caribbean Sea

**DOI:** 10.7717/peerj.16583

**Published:** 2024-01-15

**Authors:** Jorge Paramo, Alfredo Rodriguez, Camilo Quintana

**Affiliations:** 1 Tropical Fisheries Science and Technology Research Group, University of Magdalena, Santa Marta, Magdalena, Colombia; 2 Doctoral Program in Marine Sciences, University of Magdalena, Santa Marta, Magdalena, Colombia

**Keywords:** Length-weight relationships, Growth, Condition factor, Deep-water crustaceans, Colombian Caribbean Sea

## Abstract

Length-weight relationships (LWR) and relative condition factor were described for species of deep-water crustaceans caught with bottom trawls in a depth range between 150 and 535 m during August and December of 2009, and March and May of 2010 in the Colombian Caribbean Sea. A linear regression was performed using the logarithmically transformed data to calculate the *a* and *b* coefficients of the LWR for 22 crustacean species corresponding to 13 families and 19 genera and three types of crustaceans (shrimp, crab, lobster). Six crustacean species showed a maximum total length greater than that reported in SeaLifeBase: *Garymunida longipes* (77.00 mm), *Eunephrops bairdii* (220.00 mm), *Metanephrops binghami* (197.46 mm), *Penaeopsis serrata* (149.00 mm), *Polycheles typhlops* (196.27 mm) and *Pleoticus robustus* (240.00 mm). A total of 11 species (50.0%) exhibited isometric growth, five species (22.7%) negative allometric and six species (27.3%) positive allometric. This study shows the first estimates of LWR for 12 species of deep-water crustaceans in the Colombian Caribbean Sea. We demonstrate for the first time that the growth parameters (intercept and slope) of the LWR varying significantly as a function of the body shape of crabs, lobsters and shrimps in deep-water crustaceans.

## Introduction

Deep-sea crustaceans support important global fisheries and are very important for the conservation of biodiversity as they support a wide variety of species (Chang et al., 2012; Boenish et al., 2022)); therefore, knowledge of population aspects is relevant for the implementation of management and conservation strategies. New potential deep-water crustacean fishing resources have been identified in the Colombian Caribbean (Paramo & Saint-Paul, 2012a; Paramo & Saint-Paul, 2012b; Paramo & Saint-Paul, 2012c). However, a potential sustainable use of those resources needs an ecosystem approach to fisheries management (EAF) that balance diverse societal objectives, by taking account of biotic, abiotic, and human components of ecosystems and their interactions and applying a holistic approach to fisheries management (Garcia et al., 2003; Bianchi, 2008). Deep-water crustaceans with the highest biomass in the Colombian Caribbean are the deep-water giant red shrimp (*Aristaeomorpha foliacea*, Risso, 1827), the royal red shrimp (*Pleoticus robustus*, Smith, 1885) (Paramo & Saint-Paul, 2012a), the pink speckled deep-water shrimp (*Penaeopsis serrata*, Bate, 1881) (Paramo & Saint-Paul, 2012b), the deep-water lobster (*Metanephrops binghami*, Boone, 1927) (Paramo & Saint-Paul, 2012c), the Squat lobster (*Agononida longipes*, Milne-Edwards, 1880) (Espitia, Paramo & Wolff, 2019) and the shrimp (*Pleosionika longipes*, Milne-Edwards, 1881) (Pérez, Paramo & Wolff, 2019). However, more scientific information is required about the life cycle, length-weight relationship and population characteristics of deep-water crustaceans, both commercial and non-commercial, before initiating a new commercial fishery. The length-weight relationships (LWR) provide information on the type of growth, the state of the species, habitat conditions and the morphometric characteristics of the species (Erzini, 1994; Gonçalves et al., 1997; Morato et al., 2001; Froese, 2006; Kampouris, Kouroupakis & Batjakas, 2020; Falsone et al., 2022). LWR and condition factor parameters are obtained from length frequency data, which are very useful for estimating biomass and comparing the life history of species between regions (Erzini, 1994; Santos et al., 2002; Froese, 2006). However, LWR parameters may vary between habitats and regions, so accurate estimation of local parameters is essential for comparative studies in stock assessment (Vaz-dos Santos & Gris, 2016; Sousa, Vasconcelos & Riera, 2020). Additionally, the condition factor based on LWR data is relevant for examining the welfare of populations (Froese, 2006; Koushlesh et al., 2017; Jisr et al., 2018). In data-limited fisheries, often lack sufficient biological information to infer the status of the fish stocks, generally based only on catches, indices, or size classes (Shephard et al., 2020). Another issue of limited-data for fisheries management is the insufficient data the information is insufficient to produce quantitative stock assessment (Dowling et al., 2011), to determine biological reference points for fisheries management (Dowling et al., 2011; Edwards, 2015). However, despite their importance, information on LWRs and condition factor are only available for a limited number of species (Froese, 2006) and are very scarce in data-limited fisheries from the Colombian Caribbean. In this way, in this work the LWR and the relative condition factor of 22 species of deep-water crustaceans in the Colombian Caribbean Sea were determined, with the purpose of contributing to the knowledge of the biology of deep-water crustaceans.

## Material and Methods

### Study area and sampling

**Figure 1:**
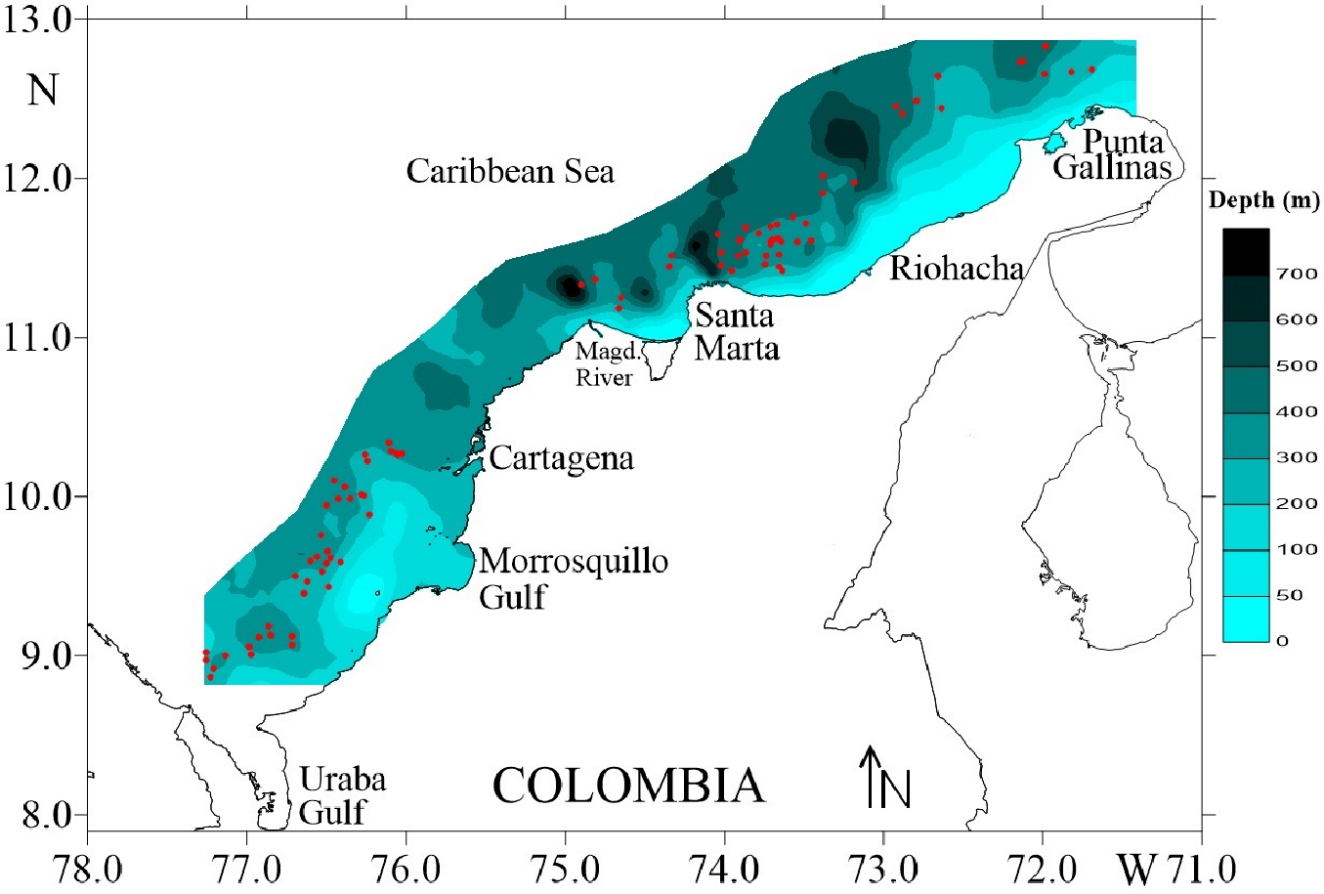
Study area in the Colombian Caribbean Sea. Red circles indicate the sampled stations. Own elaboration by CITEPT-Unimagdalena research group.

Four surveys were carried out during August and December of 2009, and March and May of 2010, on deep-water ecosystems of the Colombian Caribbean Sea. Biological data of deep-sea crustaceans were collected by trawling at depths 150–535 m. Sampling was carried out on a commercial fishing vessel using a Furuno 1150 echo sounder (28 kHz transducer) (Fig. 1). A trawl net was used with a cod-end mesh size of 44.5 mm from knot to knot. The duration of each trawl on average was 30 min and the distance travelled was calculated using a Global Positioning System (GPS) Garmin MAP 76CSx.

The captured individuals were identified to the lowest possible taxonomic level using specialized guides and literature for each taxon (Carpenter, 2002). The total length of each individual was measured with a digital calliper with a precision of 0.01 mm, and the total weight was estimated using an analytical balance with an accuracy of 0.01 mg.

The permit, care and use of experimental animals complied with Autoridad Nacional de Licencias Ambientales de Colombia (ANLA), animal welfare laws, guidelines and policies as approved by Universidad del Magdalena reference number 1293-2013.

### Statistical analysis

The information of the parameters of the LWR for each one of the crustacean species was consulted in the SeaLifeBase (https://www.sealifebase.ca/) 06/2023 data base (Palomares & Pauly, 2023) and according to information available in the scientific literature. The LWR parameters of the crustacean species were determined by applying the following allometric Eq. (1) (Keys, 1928; Le Cren, 1951; Froese, 2006):

(1) \begin{eqnarray*}W=a{L}^{b}\end{eqnarray*}

where *W* is the total body weight (g), *L* the total length (mm), for shrimp and lobster was measured from the tip of the rostrum to the end of the telson, while for crabs the width of the carapace was measured end to end of the lateral spines; *a* (intercept) y *b* (slope) are the estimated parameters applying the linear regression model with the log-transformed data (natural logarithm) according to the following Eq. (2):

(2) \begin{eqnarray*}\log \nolimits {W}_{i}=\log \nolimits a+b\log \nolimits {L}_{i}+{\epsilon }_{i}.\end{eqnarray*}

The corrected back-transformed predicted value of the response variable was calculated by multiplying the back-transformed predicted value by the correction factor (*cf*), where *RSE* is the residual standard error and *log*_*e*_ is used to adjust for the base of the natural logarithm (Ogle, 2016):

(3) \begin{eqnarray*}cf=e \frac{{ \left[ lo{g}_{e}RSE \right] }^{2}}{2} \end{eqnarray*}

To evaluate the type of isometric growth if *b* = 3.0, negative allometric if *b* < 3.0 and positive allometric if *b* > 3.0, a t-student test was used to determine significant differences from the estimated value of b and its 95% confidence interval (C.I.) (Zar, 2010).

To evaluate the influence of body morphology on the growth parameters *a* (intercept) and *b* (slope) of the LWR of the crustacean species, a robust multiple regression model was applied with the data grouped according to the type of crustacean (shrimp, crab, lobster) (Froese, 2006).

The relative condition factor (Krel) of the evaluated crustacean was determined according to the following Eq. (3) (Le Cren, 1951; Froese, 2006):

(4) \begin{eqnarray*}{K}_{rel}= \frac{W}{a{L}^{b}} \end{eqnarray*}

where *W* is the observed total body weight (g) of the crustacean specimens and *aL*^*b*^ is the estimated weight from the length-weight relationships. A good growth state of the species was identified when the Krel value ≥ 1.0, while a species in poor growth conditions when the Krel value <1.0 (Le Cren, 1951; Jisr et al., 2018). A one-sample *t*-test was used to verify significant differences between the Krel and the expected value of Krel = 1.0 (Zar, 2010). All statistical and graphical analyses were performed in the R 4.2.3 language (R Core Team, 2023), using the *modelr*, *FSAmisc*, *moments* and *ggplot2* packages (Wickham, 2016; Komsta & Novomestky, 2022; Ogle, 2022; Wickham, 2022).

An analysis of the influence of body morphology on the intercept (a) and slope (b) parameters of the LWR was performed, and linear regression were estimated for three groups of crustaceans: crab, lobster, shrimp. In order to evaluate differences in linear relationships between groups (crab, lobster, shrimp), an analysis of covariance was performed (ANCOVA) once the assumptions of homoscedasticity of the slopes (parallelism) were met with the data transformed into logarithm (Zar, 2010).

## Results

A total of 22 crustacean species belonging to 13 families were analyzed, of which the Munididae, Nephropidae and Pandalidae families were the most representative with four, three and three species, respectively (Table 1). Regarding body shape, 10 species showed a shrimp body shape, nine lobsters and three crabs. The most abundant species were *Penaeopsis serrata* (1,714 specimens), *Garymunida longipes* (1099 specimens), *Pleoticus robustus* (930 specimens), *Aristaeomorpha foliacea* (799 specimens), *Glyphocrangon neglecta* (688 specimens), *Metanephrops binghami* (590 specimens), *Achelous spinicarpus* (358 specimens), *Plesionika longipes* (329 specimens), *Glyphocrangon longleyi* (286 specimens) and *Solenocera acuminata* (166 specimens), belonging to families Penaeidae, Munididae, Solenoceridae, Aristeidae, Glyphocrangonidae, Nephropidae, Portunidae, Pandalidae, Glyphocrangonidae and Solenoceridae, respectively (Table 1). The sizes of the shrimps ranged from 31.79 mm of the pink speckled shrimp (*Penaeopsis serrata*) to 240 mm of the Royal red shrimp (*Pleoticus robustus*), crabs ranged from 13.59 mm of the crab (*Achelous spinicarpus*) to 68.69 mm of the five spine purse crab (*Myropsis quinquespinosa*) and lobsters ranged from 18.33 mm of the Squat lobster (*Antillimunida evermanni*) to 220.00 mm of the Red lobster (*Eunephrops bairdii*).

**Table 1:** Descriptive statistics for the 22 deep-sea crustaceans in Colombian Caribbean Sea.

**Family**	**Species**	**Author**	**N**	**Body shape**	**Total length (mm) Mean ± SD (Range)**	**Total weight (g) Mean ± SD (Range)**
Aristeidae	*Aristaeomorpha foliacea*	(Risso, 1827)	799	Shrimp	151.89 ± 28.35 (72.53–225.00)	22.84 ± 12.23 (2.00–57.20)	
Calappidae	*Acanthocarpus alexandri*	Stimpson, 1871	12	Crab	38.83 ± 8.07 (25.54–50.92)	32.89 ± 20.97 (5.90–76.50)	
Crangonidae	*Parapontocaris vicina*	(Dardeau & Heard, 1983)	73	Shrimp	78.96 ± 11.51 (54.75–110.12)	4.99 ± 2.18 (1.50–9.40)	
Glyphocrangonidae	*Glyphocrangon longleyi*	Schmitt, 1931	286	Shrimp	82.18 ± 15.38 (54.41–125.00)	5.76 ± 5.12 (0.70–25.40)	
	*Glyphocrangon neglecta*	Faxon, 1896	688	Shrimp	70.09 ± 8.00 (35.69–116.00)	2.68 ± 2.18 (2.00–57.20)	
Leucosiidae	*Myropsis quinquespinosa*	Stimpson, 1871	45	Crab	45.80 ± 10.77 (26.89–68.69)	36.67 ± 26.86 (5.80–117.90)	
Munididae	*Antillimunida evermanni*	(Benedict, 1901)	69	Lobster	46.34 ± 7.56 (18.33–73.26)	4.77 ± 2.98 (0.30–17.90)	
	*Antillimunida flinti*	(Benedict, 1902)	12	Lobster	48.52 ± 6.51 (36.45–56.41)	4.63 ± 1.47 (1.90–6.20)	
	*Babamunida forceps*	(A. Milne Edwards, 1880)	18	Lobster	53.67 ± 9.40 (35.39–67.34)	5.29 ± 2.64 (1.30–10.50)	
	*Garymunida longipes*	(A. Milne Edwards, 1880)	1099	Lobster	45.09 ± 6.49 (20.66–**77.00**)	3.77 ± 1.45 (0.39–11.35)	
Munidopsidae	*Munidopsis riveroi*	Chace, 1939	16	Lobster	41.49 ± 7.99 (31.42–56.30)	3.30 ± 1.99 (1.50–8.40)	
Nephropidae	*Eunephrops bairdii*	Smith, 1885	107	Lobster	126.72 ± 34.43 (53.12–**220.00**)	38.64 ± 37.41 (1.50–218.10)	
	*Metanephrops binghami*	(Boone, 1927)	590	Lobster	119.56 ± 27.43 (53.65–**197.46**)	28.65 ± 19.93 (1.70–109.34)	
	*Nephropsis aculeata*	Smith, 1881	113	Lobster	70.05 ± 15.53 (43.83–119.16)	5.32 ± 4.02 (0.90–20.00)	
Pandalidae	*Heterocarpus ensifer*	A. Milne-Edwards, 1881	147	Shrimp	100.37 ± 14.81 (63.43–130.30)	7.60 ± 3.35 (1.50–16.60)	
	*Plesionika edwardsii*	(Brandt, 1851)	46	Shrimp	109.91 ± 23.22 (60.98–162.00)	4.25 ± 2.31 (0.70–10.39)	
	*Plesionika longipes*	(A. Milne-Edwards, 1881)	329	Shrimp	132.62 ± 20.72 (70.87–203.00)	9.01 ± 4.26 (1.30–36.20)	
Penaeidae	*Penaeopsis serrata*	Spence Bate, 1881	1714	Shrimp	104.55 ± 16.66 (31.79–**149.00**)	5.99 ± 2.69 (0.29–16.30)	
Polychelidae	*Polycheles typhlops*	Heller, 1862	156	Lobster	87.94 ± 21.03 (44.00–**196.27**)	13.63 ± 11.64 (1.00–94.41)	
Portunidae	*Achelous spinicarpus*	Stimpson, 1871	358	Crab	24.72 ± 4.83 (13.59–50.19)	8.55 ± 5.45 (1.30–43.80)	
Solenoceridae	*Pleoticus robustus*	(Smith, 1885)	930	Shrimp	162.97 ± 30.02 (62.00–**240.00**)	32.73 ± 18.22 (1.09–96.10)	
	*Solenocera acuminata*	Pérez Farfante & Bullis, 1973	166	Shrimp	103.00 ± 18.85 (57.00–199.00)	12.49 ± 8.13 (1.50–71.90)	

**Notes.**

N sample size SD standard deviation Bold maximum total length longer than in SeaLifeBase

Species with a maximum total length greater than that reported in SeaLifeBase were *Garymunida longipes* (77.00 mm), *Eunephrops bairdii* (220.00 mm), *Metanephrops binghami* (197.46 mm), *Penaeopsis serrata* (149.00 mm), *Polycheles typhlops* (196.27 mm) and *Pleoticus robustus* (240.00 mm) (Table 1). Linear regressions were significant for all species (*p* < 0.05), with coefficients of determination (r^2^) between 0.73 and 0.96, except for the lobsters *Garymunida longipes* and *Antillimunida flinti* with the lowest values of 0.56 and 0.68, respectively (Table 2). The intercept of the linear regression (*a*) showed a range of values for shrimps between 6.5326E-08 and 4.4700E-05, for lobsters between 3.5531E-06 and 5.6059E-04 and for crabs between 1.2717E-04 and 5.9070E-04. The slope parameters (*b*) were for shrimps between 2.41 and 4.09, for lobsters between 2.30 and 3.29 and for crabs between 2.95 and 3.31 (Table 2).

**Table 2:** Length-weight relationships (LWR) for the 22 deep-water crustaceans in Colombian Caribbean Sea.

**Family**	**Species**	**Body shape**	**Relationship parameters**							**Krel** ** Mean ± SD**	***t*-test**
			**a**	**95% C.I. of a**	**b**	**95% C.I. of b**	**r** ^2^	**t-student**	**Growth type**		
Aristeidae	*Aristaeomorpha foliacea*	Shrimp	1.1571E−05	7.3505E−06–1.8215E−05	2.86	2.77–2.95	0.83	0.00	Allometric (-)	1.03 ± 0.28	1.00
Calappidae	*Acanthocarpus alexandri* (*ϕ*)	Crab	1.5821E−04	1.8009E−05–1.3899E−03	3.31	2.71–3.90	0.94	0.28	Isometric	1.02 ± 0.18	0.61
Crangonidae	*Parapontocaris vicina* (*ϕ*)(Δ)	Shrimp	1.2994E−05	3.5077E−06–4.8132E−05	2.93	2.63–3.23	0.84	0.63	Isometric	1.02 ± 0.19	0.78
Glyphocrangonidae	*Glyphocrangon longleyi*	Shrimp	6.5326E−08	3.7290E−08–1.1444E−07	4.09	3.97–4.22	0.93	0.00	Allometric (+)	1.02 ± 0.20	0.95
	*Glyphocrangon neglecta* (*ϕ*)	Shrimp	4.4206E−07	2.4300E−07–8.0419E−07	3.65	3.51–3.79	0.79	0.00	Allometric (+)	1.02 ± 0.32	0.98
Leucosiidae	*Myropsis quinquespinosa* (*ϕ*)(Δ)	Crab	1.2717E−04	4.3470E−05–3.7200E−04	3.23	2.95–3.52	0.93	0.10	Isometric	1.03 ± 0.23	0.77
Munididae	*Antillimunida evermanni* (*ϕ*)(Δ)	Lobster	5.5929E−05	1.7366E−05–1.8012E−04	2.93	2.63–3.24	0.85	0.66	Isometric	1.02 ± 0.23	0.81
	*Antillimunida flinti* (*ϕ*)(Δ)	Lobster	4.1815E−04	4.7411E−06–3.6880E−02	2.39	1.23–3.54	0.68	0.27	Isometric	1.02 ± 0.24	0.64
	*Babamunida forceps* (*ϕ*)(Δ)	Lobster	4.0042E−05	2.0311E−06–7.8940E−04	2.93	2.18–3.69	0.81	0.86	Isometric	1.03 ± 0.24	0.70
	*Garymunida longipes* (*ϕ*)	Lobster	5.6059E−04	3.5499E−04–8.8527E−04	2.30	2.18–2.42	0.56	0.00	Allometric (-)	1.04 ± 0.29	1.00
Munidopsidae	*Munidopsis riveroi* (*ϕ*)(Δ)	Lobster	9.2196E−05	1.4943E−05–5.6885E−04	2.79	2.30–3.28	0.91	0.37	Isometric	1.01 ± 0.15	0.62
Nephropidae	*Eunephrops bairdii* (*ϕ*)(Δ)	Lobster	3.5531E−06	1.9132E−06–6.5988E−06	3.29	3.16–3.42	0.96	0.00	Allometric (+)	1.02 ± 0.18	0.84
	*Metanephrops binghami* (*ϕ*)	Lobster	3.8607E−06	2.7855E−06–5.3509E−06	3.27	3.20–3.33	0.94	0.00	Allometric (+)	1.02 ± 0.19	0.99
	*Nephropsis aculeata* (Δ)	Lobster	1.0125E−05	4.6940E−06–2.1841E−05	3.06	2.88–3.24	0.91	0.50	Isometric	1.02 ± 0.18	0.86
Pandalidae	*Heterocarpus ensifer* (*ϕ*)(Δ)	Shrimp	2.3680E−06	1.0187E−06–5.5042E−06	3.23	3.05–3.42	0.89	0.01	Allometric (+)	1.02 ± 0.17	0.86
	*Plesionika edwardsii* (Δ)	Shrimp	4.4700E−05	5.4415E−06–3.6720E−04	2.41	1.97–2.86	0.73	0.01	Allometric (-)	1.06 ± 0.39	0.84
	*Plesionika longipes* (*ϕ*)(Δ)	Shrimp	8.2265E−06	4.0646E−06–1.6650E−05	2.83	2.68–2.97	0.82	0.02	Allometric (-)	1.02 ± 0.23	0.97
Penaeidae	*Penaeopsis serrata* (*ϕ*)	Shrimp	3.6517E−05	2.7011E−05–4.9370E−05	2.57	2.50–2.63	0.78	0.00	Allometric (-)	1.03 ± 0.22	1.00
Polychelidae	*Polycheles typhlops* (Δ)	Lobster	4.2898E−05	1.4162E−05–1.2994E−04	2.79	2.54–3.04	0.76	0.09	Isometric	1.06 ± 0.42	0.97
Portunidae	*Achelous spinicarpus* (*ϕ*)	Crab	5.9070E−04	4.4436E−04–7.8523E−04	2.95	2.86–3.04	0.92	0.26	Isometric	1.01 ± 0.17	0.94
Solenoceridae	*Pleoticus robustus* (*ϕ*)	Shrimp	3.6061E−06	2.9410E−06–4.4217E−06	3.12	3.08–3.16	0.96	0.00	Allometric (+)	1.01 ± 0.13	0.97
	*Solenocera acuminata* (*ϕ*)	Shrimp	1.1801E−05	5.1777E−06–2.6899E−05	2.97	2.79–3.15	0.87	0.72	Isometric	1.02 ± 0.20	0.91

**Notes.**

a intercept b slope C.I. lower and upper confidence (95%) r^2^ determination coefficient *t*-student *p*-value of *t*-student I isometric growth A(-) negative allometric growth A(+) positive allometric growth Krel relative condition factor *t*-test *p*-value of one-sample *t*-test (*ϕ*) No available data of LWR in SeaLifeBase (Δ) First report of the LWR in the Colombian Caribbean Sea

According to growth type, 11 species (50.0%) showed isometric growth (*b* = 3), of which two species were shrimps, six were lobsters and three were crabs. However, six species (27.3%) showed positive allometric growth (b>3), of which four species were shrimps and two species were lobsters. Nevertheless, five species (22.7%) showed negative allometric growth (b<3), of which four were shrimps and one was lobster. It is important to highlight that 17 species evaluated do not have LWR values in SeaLifeBase (https://www.sealifebase.ca/) and the first LWR report for 12 species of deep-water crustaceans in the Colombian Caribbean Sea is shown (Table 2).

**Figure 2:**
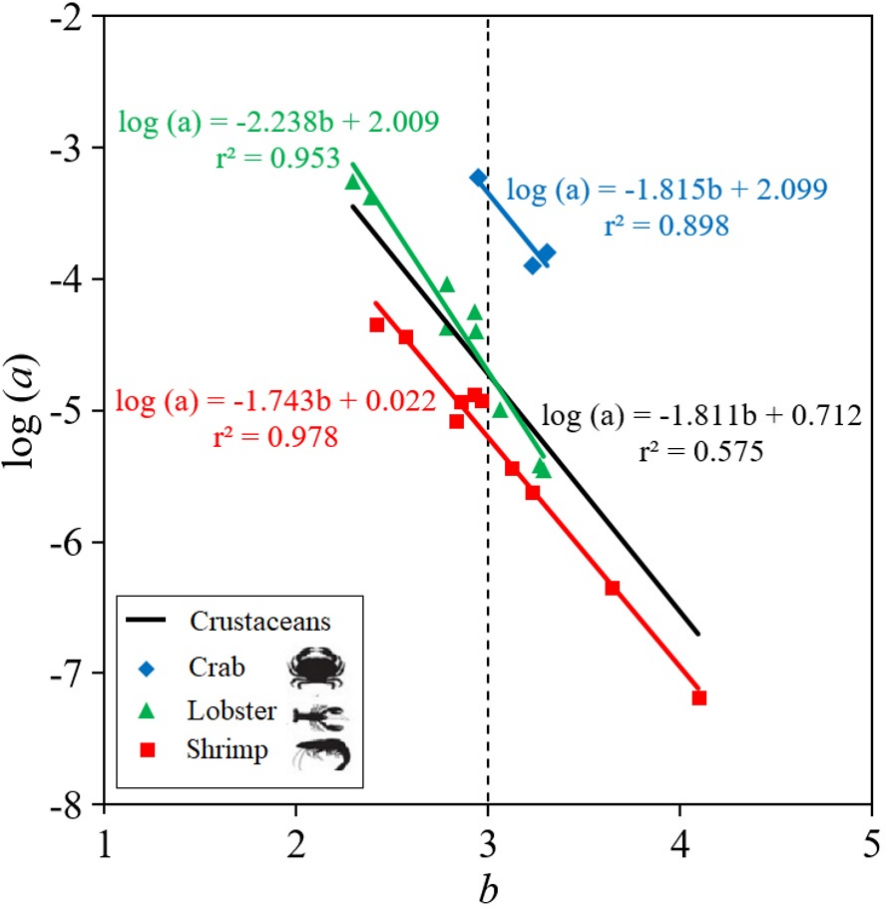
Scatter plot of mean log *a* over mean *b* for 22 deep-water (three of types of crustaceans: shrimp, crab, lobster) in Colombian Caribbean Sea. All crustaceans: black line; crab: blue rhombus; Lobster: green triangle; shrimp: red square.

The parameters of the LWR linear regression, the intercept log (*a*) and slope (*b*), are highly dependent on the body shape of the crustacean species (Fig. 2). Thus, shrimp species tend to be positive allometric (40.0%), negative allometric (40.0%) and only 20.0% were isometric, while lobster and crab species tend to be isometric, with 66.7% and 100%, respectively, although 22.2% and 11.1% of the lobsters were positive allometric and negative allometric, respectively (Table 2). The results of the ANCOVA revealed that there were significant differences ( *p* < 0.01) between the slopes (*b*) of body shape of crabs, lobsters and shrimps in the LWR relationship. When the LWR linear regression was made between the intercept log(a) and the slope b of all crustaceans, the *r*^2^ = 0.575 was low. The slope was larger in lobsters (b = −2.238) and smaller in crabs (b = −1.815) and shrimps (b = −1.743) (Fig. 2).

**Figure 3:**
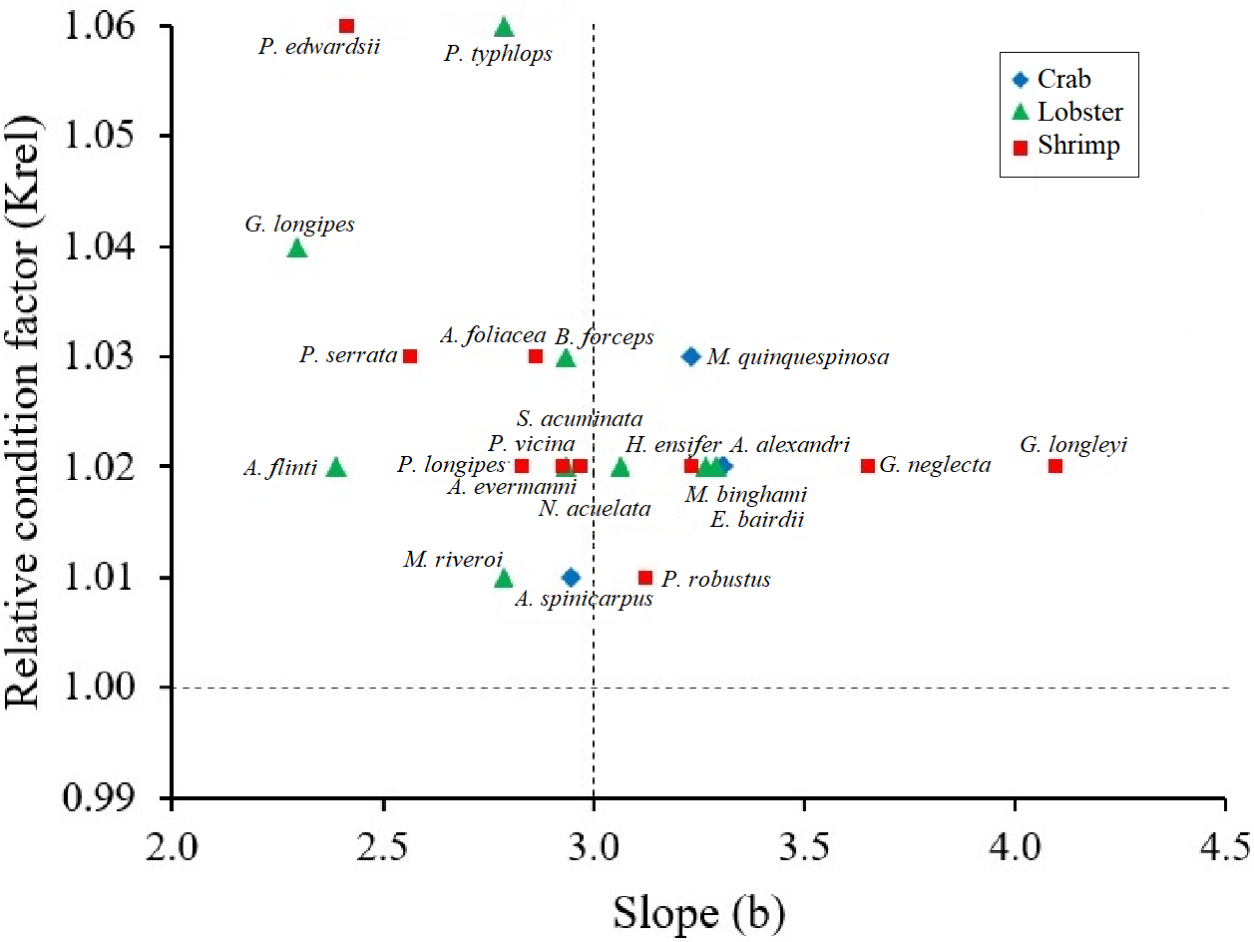
Scatter of the relationship between the relative condition factor (Krel) and the body condition factor (b) in type of crustaceans (shrimp, crab, lobster). All crustaceans: black line; crab: blue rhombus; Lobster: green triangle; shrimp: red square.

Krel values were greater than 1 in all crustacean species, confirming healthy conditions. The species that showed larger Krel were the shrimp *P. edwardsii* and the lobster *P. typhlops* and *G. longipes* (Fig. 3). Although there are no significant differences (Kruskal-Wallis test, *p*-value = 0.1586) between the Krel, crabs had lowest Krel values on average 1.02, while lobsters and shrimps had 1.03 (Table 2).

## Discussion

From the point of view of sustainable fisheries management, it is relevant to know the size structure of the populations, especially in these populations of deep-water crustaceans in the Colombian Caribbean that have not been commercially exploited and the growth type and relative condition factor can be considered sustainable biological reference points. In this way, when analyzing the sizes, the demographic parameters of a population can be described in relation to the fishing pressure (Lizárraga-Cubedo, Pierce & Santos, 2008). In addition, the patterns of morphometric variation indicate differences in growth, since the shape of the body is the product of ontogeny, that is, structural changes in the development of the organism, which is very important to implement efficient fisheries management measures (Cadrin, 2005). Through the determination of the relationships between the size structure of the body morphology of groups of crustaceans, management measures can be implemented for the beginning of a fishery, such as: average size of the catch, selectivity in the fishing gear, type growth (allometric and isometric), relative condition factor, etc. (Barbosa-Saldaña, Díaz-Jaimes & Uribe-Alcocer, 2012). Although size is usually measured as a length, weight measurements are required in fisheries to calculate fishing yield, so it is very useful to determine morphometric relationships (King, 2007).

Fisheries science seeks the generation of new knowledge to know the aspects that favor the sustainability of marine resources, such as the analysis of length-weight and relative condition factor relationships, which can provide an important insight into the ecology of the species (Froese, 2006). Pauly (1993) mentions requirements in marine stock assessment where length-weight relationships may be needed, which include: (1) conversion of individual fish length to weight, (2) estimation of mean weight of a given length class, (3) conversion of the length growth equation to a weight growth equation, and (4) morphological comparisons between populations of the same species, or between species. For most fish species, data on length-weight relationships are available from FishBase (http://www.fishbase.org), which is a global fish information system (Froese & Pauly, 2023). However, information on crustaceans and cephalopods is available in a similar information system called SeaLifeBase (http://www.sealifebase.org), although with very little information, especially for deep-water crustaceans (Palomares & Pauly, 2023).

In the Colombian Caribbean there are very few antecedents of LWR studies in deep-water crustaceans, therefore, it is not possible to determine if there are variations of the parameter b and the type of growth of the species. However, in marine populations, variations in growth are related to factors such as ontogeny, feeding (quantity, quality and size), sex, state of maturity, health, seasonality, habitat, range of sizes and the sample size (Keys, 1928; Le Cren, 1951; Safran, 1992; Moyle & Cech Jr, 2004; Froese, Tsikliras & Stergiou, 2011; Correa-Herrera, Jiménez-Segura & Barletta, 2016).

Four species showed low numbers of specimens, the crab *Acanthocarpus alexandri* (*n* = 12) and the lobsters *Antillimunida flinti* (*n* = 12), *Munidopsis riveroi* (*n* = 16) and *Babamunida forceps* (*n* = 18) (Table 1). However, according to Froese (2006) for length-weight relationships a low number of specimens may be acceptable when species are rare, such as these deep-sea crustaceans. A total of 77.3% of the crustacean species evaluated presented a range of parameter b between 2.5 and 3.5 (Froese, 2006), indicating normal growth dimensions (Bagenal & Tesch, 1978; King, 2007), except in *Antillimunida flinti*, *Garymunida longipes* and *Plesionika edwardsii* with values of *b* < 2.5, while *Glyphocrangon longleyi* and *Glyphocrangon neglecta* with *b* > 3.5, showed a narrow size range, common in values of *b* < 2.5 or >3.5 (Carlander, 1977; Froese, 2006). Additionally, the giant red shrimp *Aristaeomorpha foliacea* showed negative allometric growth off the west coast of Sicily (Falsone et al., 2022) and in the Tyrrhenian Sea (Apostolidis & Stergiou, 2008), which agrees with our findings. However, it differs from the positive allometric growth reported for this species in the Strait of Sicily (Ragonese et al., 2004). The *Plesionika edwardsii* shrimp showed variations in the type of growth with positive allometric in females and negative allometric in males in the Spanish coast of the Western Mediterranean Sea (García-Rodriguez, Esteban & Pérez-Gil, 2000; Company & Sardá, 2000). In addition, isometric growth was reported in the lobster *Polycheles typhlops* (Company & Sardá, 2000), which also coincides with this study.

## Conclusions

We demonstrate for the first time that the growth parameters (intercept and slope) of the LWR varying significantly as a function of the body shape of crabs, lobsters and shrimps in deep-water crustaceans. Shrimp tend to have allometric growth, positive or negative, but lobster and crabs’ growth type is mainly isometric. Regarding the relative condition factor, we found that all the species evaluated had good health conditions, which may be related to the fact that in the Colombian Caribbean the deep-water ecosystem can still be considered pristine and there is currently no fishery; therefore, there is no negative impact from fishing on these deep-water crustacean species (Paramo & Saint-Paul, 2012a; Paramo & Saint-Paul, 2012b; Paramo & Saint-Paul, 2012c). The LWR parameters and the relative condition factor can be applied as simple and inexpensive indicators to implement to assess the well-being of marine populations, considering that a change in the growth conditions of the species may indicate an impact of anthropogenic origin and/or environmental. Finally, although the results of this study were obtained from mixed sexes, they are of great importance for the management of marine resources, since management and conservation regulations are not specific for each sex and can be applied to the entire population (Falsone et al., 2022).

##  Supplemental Information

10.7717/peerj.16583/supp-1 Supplemental Information 1 Length and weight of deep-water crustaceans Colombian Caribbean

## Additional Information and Declarations

### Field Study Permissions

The following information was supplied relating to field study approvals (*i.e.*, approving body and any reference numbers): The permit, care and use of experimental animals complied with Autoridad Nacional de Licencias Ambientales de Colombia (ANLA), animal welfare laws, guidelines and policies as approved by Universidad del Magdalena reference number 1293-2013.

### Data Availability

The following information was supplied regarding data availability: The raw data is available in the Supplemental File.

## References

[ref-1] Apostolidis CA, Stergiou KI, Palomares MLD, Pauly D (2008). A preliminary compilation of life-history data for Mediterranean marine invertebrates. Von Bertalanffy Growth Parameters of Non-fish Organisms. Fisheries Centre Research Reports.

[ref-2] Bagenal TB, Tesch FW, Bagenal TB (1978). Age and growth. Methods for assessment of fish production in fresh waters.

[ref-3] Barbosa-Saldaña M, Díaz-Jaimes P, Uribe-Alcocer M (2012). Variación morfológica del camarón café (*Farfantepenaeus californiensis*) en el Pacífico mexicano. Revista Mexicana de Biodiversidad.

[ref-4] Bianchi G, Bianchi G, Skjoldal HR (2008). The concept of the ecosystem approach to fisheries. The ecosystem approach to fisheries.

[ref-5] Boenish R, Kritzer JP, Kleisner K, Steneck RS, Werner KM, Zhu W, Schram F, Rader D, Cheung W, Ingles J, Tian Y, Mimikakis J (2022). The global rise of crustacean fisheries. Frontiers in Ecology and the Environment.

[ref-6] Cadrin SX, Cadrin SX, Friedland KD, Waldman JR (2005). Morphometric landmarks. Stock identification methods: applications in fishery science.

[ref-7] Carlander KD (1977). Handbook of freshwater fishery biology.

[ref-8] Carpenter KE (2002). The living marine resources of the Western Central Atlantic. Volume 1: Introduction, molluscs, crustaceans, hagfishes, sharks, batoid fishes, and chimaeras.

[ref-9] Chang YJ, Sun CL, Chen Y, Yeh SZ (2012). Modelling the growth of crustacean species. Reviews in Fish Biology and Fisheries.

[ref-10] Company JB, Sardá F (2000). Growth parameters of deep-water decapod crustaceans in the Northwestern Mediterranean Sea: a comparative approach. Marine Biology.

[ref-11] Correa-Herrera T, Jiménez-Segura LF, Barletta M (2016). Fish species from a micro–tidal delta in the Caribbean Sea. Journal of Fish Biology.

[ref-12] Dowling NA, Haddon M, Smith DC, Dichmont CM, Smith AD (2011). Harvest strategies for data-poor fisheries: a brief review of the literature.

[ref-13] Edwards CTT (2015). Review of data-poor assessment methods for New Zealand fisheries. New Zealand Fisheries Assessment Report (2015/27).

[ref-14] Erzini K (1994). An empirical study of variability in length–at–age of marine fishes. Journal of Applied Ichthyology.

[ref-15] Espitia J, Paramo J, Wolff M (2019). Spatial distribution and size structure of the squat lobster *Agononida longipes* (A. Milne Edwards, 1880) (Crustacea: Decapoda: Galatheoidea: Munididae) in the Colombian Caribbean. Latin American Journal of Aquatic Research.

[ref-16] Falsone F, Geraci ML, Scannella D, Gancitano V, Di Maio F, Sardo G, Quattrocchi F, Vitale S (2022). Length–weight relationships of 52 species from the South of Sicily (Central Mediterranean Sea). Fishes.

[ref-17] Froese R (2006). Cube law, condition factor and weight–length relationships: history, meta-analysis and recommendations. Journal of Applied Ichthyology.

[ref-18] Froese R, Pauly D (2023). FishBase.

[ref-19] Froese R, Tsikliras AC, Stergiou KI (2011). Editorial Note on Weight–Length Relations of Fishes. Acta Ichthyologica et Piscatoria.

[ref-20] Garcia SM, Zerbi A, Aliaume C, Chi T, Lasserre G (2003). The ecosystem approach to fisheries. Issues, terminology, principles, institutional foundations, implementation and outlook.

[ref-21] García-Rodriguez M, Esteban A, Pérez-Gil JL (2000). Considerations on the biology of *Plesionika edwardsi* (Brandt, 1851) (Decapoda, Caridea, Pandalidae) from experimental trap catches in the Spanish western Mediterranean Sea. Scientia Marina.

[ref-22] Gonçalves JMS, Bentes L, Lino PG, Ribeiro J, Canario AV, Erzini K (1997). Weight–length relationships for selected fish species of the small-scale demersal fisheries of the south and south-west coast of Portugal. Fisheries Research.

[ref-23] Jisr N, Younes G, Sukhn C, El-Dakdouki MH (2018). Length–weight relationships and relative condition factor of fish inhabiting the marine area of the Eastern Mediterranean city, Tripoli-Lebanon. Egyptian Journal of Aquatic Research.

[ref-24] Kampouris TE, Kouroupakis E, Batjakas IE (2020). Morphometric relationships of the global invader *Callinectes sapidus* Rathbun, 1896 (Decapoda, Brachyura, Portunidae) from Papapouli Lagoon, NWAegean Sea, Greece. With notes on its ecological preferences. Fishes.

[ref-25] Keys AB (1928). The weight–length relationship in fishes. Proceedings of the National Academy of Sciences of the United States of America.

[ref-26] King M (2007). Fisheries biology, assessment and management.

[ref-27] Komsta L, Novomestky F (2022). https://cran.r-project.org/web/packages/moments/.

[ref-28] Koushlesh SK, Sinha A, Kumari K, Borah S, Chanu TN, Baitha R, Das SK, Gogoi P, Sharma SK, Ramteke MH, Das BK (2017). Length–weight relationship and relative condition factor of five indigenous fish species from Torsa River, West Bengal, India. Journal of Applied Ichthyology.

[ref-29] Le Cren ED (1951). The length–weight relationship and seasonal cycle in gonad weight and condition in the perch (*Perca fluviatilis*). Journal of Animal Ecology.

[ref-30] Lizárraga-Cubedo HA, Pierce GJ, Santos MB, Mente E (2008). Reproduction of crustaceans in relation to fisheries. Reproductive biology of crustaceans.

[ref-31] Morato T, Afonso P, Lourinho P, Barrelieros JP, Santos RS, Nash RD (2001). Length–weight relationships for 21 coastal fish species of the Azores, north-eastern Atlantic. Fisheries Research.

[ref-32] Moyle PB, Cech Jr JJ (2004). Fishes: an introduction to ichthyology.

[ref-33] Ogle DH (2016). Introductory fisheries analyses with R.

[ref-34] Ogle DH (2022). https://github.com/droglenc/FSAmisc.

[ref-35] Palomares MLD, Pauly D (2023). SeaLifeBase.

[ref-36] Paramo J, Saint-Paul U (2012a). Deep-sea shrimps *Aristaeomorpha foliacea* and *Pleoticus robustus* (Crustacea: Penaeoidea) in the Colombian Caribbean Sea as a new potential fishing resource. Journal of the Marine Biological Association.

[ref-37] Paramo J, Saint-Paul U (2012b). Spatial structure of the pink speckled deep-sea shrimp *Penaeopsis serrata* (Bate, 1881) (Decapoda, Penaeidae) during november-december 2009 in the Colombian Caribbean Sea. Crustaceana.

[ref-38] Paramo J, Saint-Paul U (2012c). Spatial structure of deep-sea lobster (*Metanephrops binghami*) in the Colombian Caribbean Sea. Helgoland Marine Research.

[ref-39] Pauly D (1993). Editorial: Fishbyte Section. Naga, The ICLARM Quarferly.

[ref-40] Pérez D, Paramo J, Wolff M (2019). Distribution, abundance and fishing potential of mega-invertebrates in the sub-euphotic zone (150–535 m) in the Colombian Caribbean. Regional Studies in Marine Science.

[ref-41] R Core Team (2023). https://www.r-project.org.

[ref-42] Ragonese S, Andreoli MG, Bono G, Giusto GB, Rizzo P, Sinacori G (2004). Overview of the available information on demersal resources of the Strait of Sicily. MedSudMed Technical Documents.

[ref-43] Safran P (1992). Theoretical analysis of the weight–length relationship in fish juveniles. Marine Biology.

[ref-44] Shephard S, Valbo-Jorgensen J, Abadía J, Baigún C, Doria CRC, Fabré NN, Isaac VJ, Ngor PB, Ruffino ML, Funge-Smith SJ (2020). Size-based assessment of data-limited inland fish stocks—Review and applications. FAO Fisheries and Aquaculture Circular (1214).

[ref-45] Vaz-dos Santos AM, Gris B (2016). Length–weight relationships of the ichthyofauna from a coastal subtropical system: a tool for biomass estimates and ecosystem modelling. Biota Neotropica.

[ref-46] Santos MN, Gaspar MB, Vasconcelos P, Monteiro CC (2002). Weight–length relationships for 50 selected fish species of the Algarve coast (southern Portugal). Fisheries Research.

[ref-47] Sousa R, Vasconcelos J, Riera R (2020). Weight–length relationships of four intertidal mollusc species from the northeastern Atlantic Ocean and their potential for conservation. Molluscan Research.

[ref-48] Wickham H (2016). https://ggplot2.tidyverse.org.

[ref-49] Wickham H (2022). https://CRAN.R-project.org/package[balance].

[ref-50] Zar JH (2010). Biostatistical analysis.

